# Nanoparticle-enhanced CRISPR delivery: paving the path for *in vivo* tumor gene editing

**DOI:** 10.1097/MS9.0000000000004414

**Published:** 2025-11-25

**Authors:** Muhammad Khizar, Hasibullah Aminpoor, Muhammad Zaib, Qaima Ali, Hasiba Karimi

**Affiliations:** a Faculty of Medicine, Georgian American University, Manifest Medical Research Co, Tbilisi, Georgia; b Faculty of Medicine, Kabul University of Medical Sciences “Abu Ali Ibn Sina”, Kabul, Afghanistan; c Faculty of Medicine, Liaquat College of Medicine & Dentistry, Karachi, Pakistan; d Faculty of Medicine, Bezmialem Vakif University, Istanbul, Turkey

**Keywords:** cancer therapy, CRISPR Cas9, *in vivo* gene editing, lipid nanoparticles, nanoparticle delivery, precision oncology

## Abstract

Nanoparticle-based delivery systems are redefining how CRISPR/Cas technology can be used in cancer treatment. By encapsulating CRISPR components within lipid, polymeric, or inorganic nanoparticles, researchers have improved their stability, circulation time, and tumor-targeting precision. The NTLA-2001 trial demonstrated the first successful use of lipid nanoparticles for *in vivo* CRISPR delivery in humans, paving the way for potential applications in oncology. Preclinical studies have shown promising results, with efficient gene knockout and tumor suppression across multiple models. Despite these advances, barriers remain, including limited delivery to solid tumors, potential off-target effects, and inconsistent nanoparticle formulations. Global research efforts spanning the United States, China, Europe, and India are now focused on refining delivery platforms and standardizing protocols. This letter highlights current progress, ongoing challenges, and the need for transparent, globally coordinated development. Nanoparticle-enhanced CRISPR delivery has the potential to bring genetic precision therapy from the laboratory to the clinic, offering a new avenue for durable and accessible cancer care.

*To the Editor*,

The convergence of CRISPR/Cas genome editing and nanoparticle (NP) delivery technologies represents one of the most promising developments in modern oncology. The idea of directly repairing or disabling tumor-promoting genes within the human body could fundamentally change how we approach cancer therapy. Conventional treatments such as chemotherapy and radiotherapy remain effective but often fail to address the underlying genetic mutations driving tumor growth. CRISPR technology, in contrast, offers the precision to target those genetic defects, if we can deliver it safely and effectively inside the body.

The primary limitation of CRISPR in cancer therapy lies in its delivery. DNA and RNA components are rapidly degraded in circulation, while the large Cas9 protein struggles to cross cell membranes. Nanoparticles offer a solution by protecting these components and improving their stability and uptake. Lipid nanoparticles (LNPs), in particular, have shown remarkable promise. The NTLA-2001 trial provided the first clinical evidence that CRISPR could be delivered safely to human hepatocytes using LNPs, establishing a milestone for *in vivo* genome editing^[1]^. Translating this success to oncology could enable direct genetic correction within solid tumors – a goal long considered out of reach.

Worldwide, researchers are experimenting with various nanocarrier systems to improve CRISPR delivery. Lipid, polymeric, and inorganic nanoparticles can be customized to transport Cas9 mRNA, guide RNA, or ribonucleoprotein complexes. Some are functionalized with tumor-homing ligands that recognize cancer-specific surface markers, increasing selectivity while reducing systemic toxicity^[2,3]^. Others are designed to release their contents only within the acidic or hypoxic environment of tumors^[4]^. Preclinical studies have shown that such targeted systems can effectively silence oncogenes and inhibit tumor growth, with minimal immune activation^[5]^.

The clinical implications are substantial. In principle, a single infusion of NP-delivered CRISPR could silence oncogenic drivers such as KRAS or restore the function of tumor suppressor genes like TP53. Combined with surgery, radiotherapy, or checkpoint inhibitors, this approach could also reprogram the tumor microenvironment to make it more responsive to immune attack. In low- and middle-income countries, such as India and Brazil, scalable NP-CRISPR systems based on biodegradable materials could offer affordable, durable alternatives to repeated chemotherapy cycles^[6,7]^.

However, several obstacles remain before these technologies reach the clinic. Most data come from mouse models, which do not fully capture the complexity of human tumors. Nanoparticles often accumulate in the liver or spleen, raising safety concerns about off-target editing. The only completed human *in vivo* CRISPR trial so far targeted liver cells rather than solid tumors because of their natural accessibility^[1]^. Delivering nanoparticles to dense or poorly vascularized tumors, such as those in the pancreas or brain, remains a major hurdle. Moreover, inconsistent nanoparticle composition and characterization across studies make it difficult to reproduce results or compare outcomes^[8,9]^. Addressing these issues will require harmonized nanoparticle standards, large-animal testing, and early human trials that include detailed biodistribution and gene-editing analysis^[10]^.

Progress will also depend on global collaboration. The United States and China currently lead in nanoparticle-based CRISPR research, while European and Japanese teams are advancing new nanocarrier materials and imaging methods to monitor *in vivo* editing^[4,6]^. Researchers in India have made significant contributions through the development of low-cost, biodegradable nanocarriers that could expand access in resource-limited settings^[7]^. These global examples highlight the importance of transparent reporting and shared regulatory frameworks to ensure both safety and equity.

In summary, nanoparticle-enhanced CRISPR delivery stands at the forefront of precision oncology. By improving the stability, targeting, and intracellular delivery of CRISPR systems, nanoparticles could make gene correction a realistic option for treating malignancy at its genetic root. Continued progress will depend on rigorous safety studies, transparent data sharing, and coordinated international research. With sustained collaboration, NP-CRISPR technology could soon transition from experimental innovation to clinical reality, reshaping the future of cancer treatment.

Our work is in line with the TITAN Guidelines on the need for transparency in AI use in healthcare^[11]^.

## Data Availability

Not applicable.
